# Validation of an Ultraviolet Light Response Gene Signature for Predicting Prognosis in Patients with Uveal Melanoma

**DOI:** 10.3390/biom13071148

**Published:** 2023-07-19

**Authors:** Carlos A. Orozco, Alejandro Mejía-García, Marcela Ramírez, Johanna González, Luis Castro-Vega, Richard B. Kreider, Silvia Serrano, Alba Lucia Combita, Diego A. Bonilla

**Affiliations:** 1 Health and Sport Sciences Research Group, School of Health and Sport Sciences, Fundación Universitaria del Área Andina, Bogotá 111221, Colombia; 2 Professional Program in Surgical Instrumentation, School of Health and Sport Sciences, Fundación Universitaria del Área Andina, Bogotá 111221, Colombia; 3 Professional Program in Optometry, School of Health and Sport Sciences, Fundación Universitaria del Área Andina, Bogotá 111221, Colombia; 4 Technical Program in Radiology and Diagnostic Imaging, School of Health and Sport Sciences, Fundación Universitaria del Área Andina, Bogotá 111221, Colombia; 5 Grupo de Investigación Genética Molecular (GENMOL), Facultad de Ciencias Exactas y Naturales, Universidad de Antioquia, Medellín 050010, Colombia; 6 Genetics and Development of Brain Tumors Team, Paris Brain Institute (ICM), Hôpital Pitié-Salpêtrière, Inserm U 1127, CNRS UMR 7225, Sorbonne Université, 75013 Paris, France; 7 Exercise & Sport Nutrition Lab, Human Clinical Research Facility, Texas A&M University, College Station, TX 77843, USA; 8 Grupo de Investigación en Biología del Cáncer, Instituto Nacional de Cancerología de Colombia, Bogotá 111511, Colombia; 9 Grupo de Investigación Traslacional en Oncología, Instituto Nacional de Cancerología de Colombia, Bogotá 111511, Colombia; 10 School of Medicine, Microbiology Department, Universidad Nacional de Colombia, Bogotá 111321, Colombia; 11 Research Division, Dynamical Business & Science Society—DBSS International SAS, Bogotá 110311, Colombia; 12 Research Group in Physical Activity, Sports and Health Sciences (GICAFS), Universidad de Córdoba, Montería 230002, Colombia

**Keywords:** uveal melanoma, gene expression signatures, prognostic factors, bioinformatics

## Abstract

Uveal melanoma (UVM) is a highly aggressive ocular cancer with limited therapeutic options and poor prognosis particularly for patients with liver metastasis. As such, the identification of new prognostic biomarkers is critical for developing effective treatment strategies. In this study, we aimed to investigate the potential of an ultraviolet light response gene signature to predict the prognosis of UVM patients. Our approach involved the development of a prognostic model based on genes associated with the cellular response to UV light. By employing this model, we generated risk scores to stratify patients into high- and low-risk groups. Furthermore, we conducted differential expression analysis between these two groups and explored the estimation of immune infiltration. To validate our findings, we applied our methodology to an independent UVM cohort. Through our study, we introduced a novel survival prediction tool and shed light on the underlying cellular processes within UVM tumors, emphasizing the involvement of immune subsets in tumor progression.

## 1. Introduction

Uveal melanoma (UVM) is a rare and aggressive cancer that arises from the pigmented cells of the eye. Although UVM accounts for only 5% of all melanomas, it is the most common primary intraocular tumor in adults [1]. While the majority of UVM patients are diagnosed with primary tumors, approximately 50% of them develop metastatic disease, which is associated with poor survival rates due to the lack of effective treatment options [2]. Currently, the main treatment for UVM includes resection, enucleation, and radiation therapies. However, one of the major clinical challenges in UVM metastatic treatment is the lack of effective systemic therapies. Although several treatments have been explored, such as chemotherapy, immunotherapy, and targeted therapy, none of them have shown the expected clinical benefit in randomized controlled trials [3]. Therefore, the development of novel and effective therapies for UVM remains an important unmet medical need.

The clinical challenges of UVM are complex and multifaceted. In addition, this tumor is highly heterogeneous, and its molecular and genetic characteristics vary widely among patients [4]. Prognostic gene expression signatures have emerged as a promising tool to predict the risk of UVM metastasis and guide treatment decisions. Multiple studies have identified gene expression profiles that correlate with UVM metastasis and overall survival [5,6]. These gene signatures can be used to classify UVM patients into low- and high-risk groups, with the high-risk group having a significantly higher risk of developing metastasis and a worse prognosis. While gene expression profiling has shown promise in predicting UVM prognosis, it is important to note that these gene signatures are still a prognostic tool and not a diagnostic test [7].

Ultraviolet (UV) radiation is a well-known risk factor for cutaneous melanoma, and recent studies have suggested that it may also play a role in UVM development. However, epidemiological and genetic data, as well as the optical properties of the ocular media, lead to controversial conclusions concerning the involvement of solar UV radiation as a risk factor for uveal melanoma [8]. While some studies [9], reported inconclusive findings and the absence of a clear association between UV radiation exposure and UVM, other investigations [10], identified potential links between UVM and factors such as eye color and UV radiation exposition. UV-induced DNA damage is considered a critical event in cutaneous melanoma UVM pathogenesis, which may lead to the activation of oncogenes and/or inactivation of tumor suppressor genes, ultimately leading to tumor initiation and progression [11]. Nonetheless, genetic analyses seem to validate the influence of UV light as a carcinogen responsible for the development of uveal melanomas. UV hallmark mutations (CpG → TpA) are found in the codon 183 of *GNAQ* and *GNA11* and in the *RAC1* gene. This mutational homology can be interpreted as a clue as to the implication of UV light in the etiology of uveal melanoma [8].

These contrasting findings highlight the need for additional studies to elucidate the role of UV radiation as a risk. Therefore, in this study, we propose the hypothesis that identifying and characterizing genes involved in the UV light response could offer valuable insights into the mechanisms driving UVM tumorigenesis. In this study, based on a set of HALLMARK_UV_RESPONSE_UP genes from the Molecular Signatures Database (MSigDB), which represent gene expression patterns related to the response to cellular UV radiation, we conducted a bioinformatic analysis to identify a gene signature indicative of UV light response in UVM patients and we evaluated their use as a prognostic tool. Our findings may offer novel insights into the diagnosis, prognosis, and management of patients with UVM. Nonetheless, it is important to note that clinical and histopathologic data should be used in conjunction with these gene signatures to make informed treatment decisions.

## 2. Materials and Methods

### 2.1. Data Resources

The RNA sequencing data UVM were acquired from The Cancer Genome Atlas (TCGA) (database (https://portal.gdc.cancer.gov/, accessed on 8 December 2022). The gene expression quantification method used was HTSeq (high-throughput sequencing), and the unit of measurement for the gene expression dataset was FPKM-UQ (fragments per kilobase million with upper quartile normalization). The expression values were further transformed using the logarithm base 2, following the unit “log_2_(fpkm-uq + 1)”. The FPKM-UQ unit considers gene length and read counts from RNA sequencing data to estimate gene expression levels. The data selection criteria consisted of 80 UVM patients diagnosed with UVM who had available expression and clinical information.

### 2.2. Gene Prioritization

To identify genes associated with the cellular UV light response, a set of 158 genes was obtained from the Hallmark_UV_Response_UP gene signature, which is available in Molecular Signatures Database (MSigDB) (http://www.gsea-msigdb.org/gsea/index.jsp, accessed on 17 December 2022). The HALLMARK_UV_RESPONSE_UP gene set used in our study is derived from MSigDB, a well-established resource for gene set enrichment analysis. The MSigDB includes a diverse collection of over 10,000 gene sets representing various biological processes and diseases. To address redundancy and heterogeneity, the MSigDB employs a refinement process called “hallmarks,” which involves clustering and consensus methods to determine stable partitions. The refined gene sets undergo iterative refinement and independent validation. The HALLMARK_UV_RESPONSE_UP gene set specifically captures coherent expression patterns related to cellular UV radiation response. These hallmarks have undergone meticulous generation and refinement to provide more concise and reliable inputs for gene set enrichment analysis [12].

### 2.3. Ultraviolet Light Response Prognostic Model

A univariate Cox regression analysis was performed using the 158 UV light response signature genes to identify potentially prognostic genes in the UVM TCGA cohort. The survival module from the Tumor Immune Estimation Resource (TIMER) tool (http://timer.comp-genomics.org/, accessed on 17 December 2022) was utilized for this purpose. Genes showing significant correlation with UVM patient survival were selected for the final prognostic model. We utilized a ridge regression approach, implemented through the R package “glmnet,” to stratify risk based on gene expression data. A subset of genes was obtained, and their corresponding coefficients were determined to calculate the risk score. The gene expression of each gene from the defined model was multiplied by the ridge regression coefficient calculated for that gene in the final model. The low- and high-risk groups were established using the median value of the risk scores as the threshold. Patients below the median were classified as the low-risk group, while those at or above the median were classified as the high-risk group. This methodology facilitated the quantification of risk based on gene expression levels, resulting in the classification of 40 UVM patients into the high-risk group and the remaining 40 into the low-risk group.

To evaluate the prognostic value of the established prognostic model, a survival analysis was conducted using the UCSC XENA browser (http://xena.ucsc.edu/, accessed on 17 December 2022) with the high-risk and low-risk patient groups [13]. Additionally, receiver operating characteristic (ROC) curves were used to assess the accuracy of the model.

### 2.4. Analysis of Clinicopathological Features in Relation to Risk Groups

To assess the potential association between clinicopathological features of UVM and the low-risk or high-risk groups, we constructed frequency distributions using the clinical data of the 80 UVM patients. The statistical software SPSS v21.0 (IBM Corp., Armonk, NY, USA) was employed for this analysis.

### 2.5. Functional Enrichment

To compare the gene expression levels between UVM patients with low-risk scores and high-risk scores, we performed a differential expression analysis using the UCSC XENA browser. The gene expression data were normalized using the log transformation method to ensure suitable data distribution for analysis. The differential expression analysis was conducted using the limma_voom method, incorporating precision weight estimation based on the observed mean-variance relationship in the data. A significance threshold of 0.05 was applied to identify genes with a statistically significant difference in expression, controlling the false discovery rate (FDR). Additionally, a log fold change threshold of 1.5 was set to identify genes with substantial changes in expression levels between the two groups. Then, using as an input the list of the top 100 Differentially Expressed Genes (DEGs) overexpressed in the high-risk group we conducted Gene Ontology (GO) and Kyoto Encyclopedia of Genes and Genomes (KEGG) enrichment using the Enrichr web tool (https://maayanlab.cloud/Enrichr/, accessed on 10 November 2022) [14]. The enriched functional analysis results were visualized using the “SRplot” R package, which generated Enrichment Bubble plots and volcano plots.

### 2.6. Gene Set Enrichment Analysis

We analyzed RNA sequencing data from 80 UVM patients obtained from the TCGA project. We conducted gene set enrichment analysis (GSEA) [15] using all the hallmark gene sets from MSigDB. The enrichment scores were calculated to assess the overrepresentation of gene sets within the dataset. Permutation testing was performed to determine the statistical significance of the enrichment scores, and the FDR was used to control for multiple hypothesis testing. Additionally, we employed principal component analysis (PCA) for data visualization and generated classical GSEA plots to visualize the enrichment scores.

### 2.7. Analysis of the Immune Microenvironment Infiltration

To calculate immune and stromal cell infiltration for each of the 80 UVM samples from the TCGA, we used the R package “xCell” [16]. The analysis was conducted using the website (https://xcell.ucsf.edu/ accessed on 10 November 2022). We evaluated the differential expression of immune and stromal cells’ infiltration between the high-risk and low-risk UVM patients, with a significance level of *p* < 0.05.

### 2.8. Association of Gene Expression and Clinicopathological Features

We utilized the publicly available R2 platform (https://hgserver1.amc.nl/cgi-bin/r2/main.cgi, accessed on 3 January 2023) to investigate potential associations between gene expression levels from the genes in our model and clinical traits in the 80 patients from the TCGA UVM cohort.

### 2.9. Validation Cohort

To validate our prognostic model, we utilized transcriptomic information from a cohort of 63 uveal melanoma patients, including their molecular profiles derived from gene expression microarrays performed on enucleated primary tumors [17]. The analysis involved the use of Affymetrix U133plus2 Arrays to analyze the transcriptomes of the 63 UVM patients.

### 2.10. Statistical Analysis

The frequency of clinicopathological variables between the high-risk and low-risk UVM groups was compared using the chi-square or Fisher exact tests. The normality assumptions for continuous data were evaluated using the Kolmogorov–Smirnov test. To assess differences in survival based on gene expression or between the low-risk and high-risk groups, the log-rank test was conducted. Differences in immune and stromal cell infiltration, between the high-risk and low-risk UVM groups were calculated using the Mann–Whitney U test. A *p*-value of < 0.05 was considered statistically significant for all the comparisons using either SPSS v21.0 (IBM Corp., Armonk, NY, USA) or R software (v4.0.2, https://www.r-project.org/, accessed on 3 January 2023).

## 3. Results

### 3.1. Identification of UV Response-Related Genes with Prognostic Potential in Uveal Melanoma

**Figure 1:**
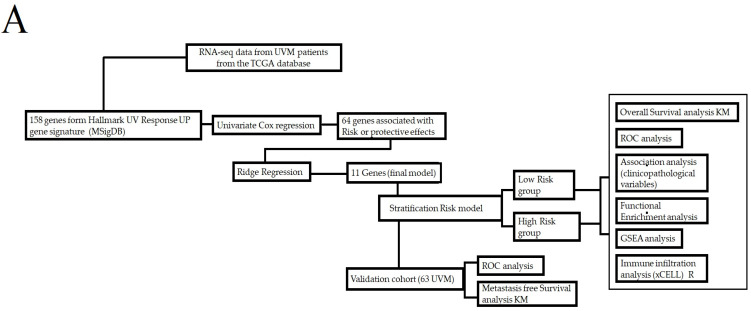

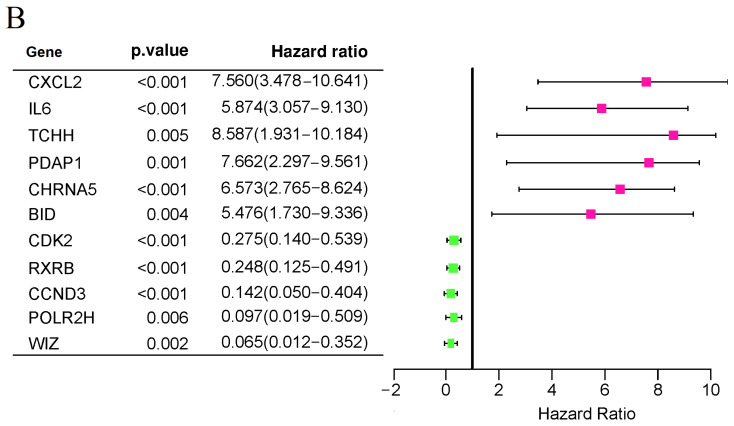
(**A**) The flowchart provides a comprehensive summary of the main methods employed in this study. (**B**) The forest plot depicts the prognostic model for UVM patients, illustrating hazard ratios (HR) and their corresponding 95% confidence intervals. Genes with protective effects are represented by green boxes, while genes associated with risk effects are represented by pink boxes.

The study flow diagram is presented in Figure 1A. Initially, a univariate Cox model was utilized to analyze 158 genes associated with UV light response [12]. This analysis aimed to identify UV response genes that could potentially impact the prognosis of UVM patients. Among the initial pool of 158 genes, 61 genes demonstrated prognostic potential. The analysis was conducted on a sample of 80 UVM patients obtained from the TCGA project. The results, including the hazard ratio (HR) and its corresponding 95% confidence interval, can be found in Supplementary Table S1. To further refine the gene list for constructing the prognostic signature, we utilized a Ridge regression model. As a result, 11 UV response genes were included in the final prognostic model, which is listed in Figure 1B.

### 3.2. Characteristics of Population

The demographic and clinical features of the 80 UVM patients from the TCGA project are detailed in Table 1. To compare patients at high-risk or low-risk, we analyzed the distribution of clinicopathological characteristics within the UVM TCGA cohort. The analysis showed that the high-risk group exhibited a higher frequency of cytogenetic abnormalities and a greater number of patients with residual tumors after treatment, in comparison to the low-risk group (*p* < 0.05).

### 3.3. Survival and Clinicopathological Features Associated with Defined UV Response Risk Scores

**Figure 2:**
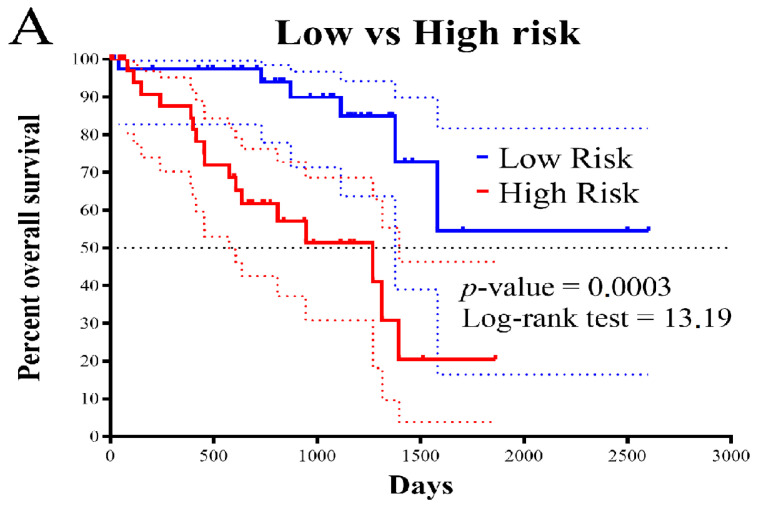

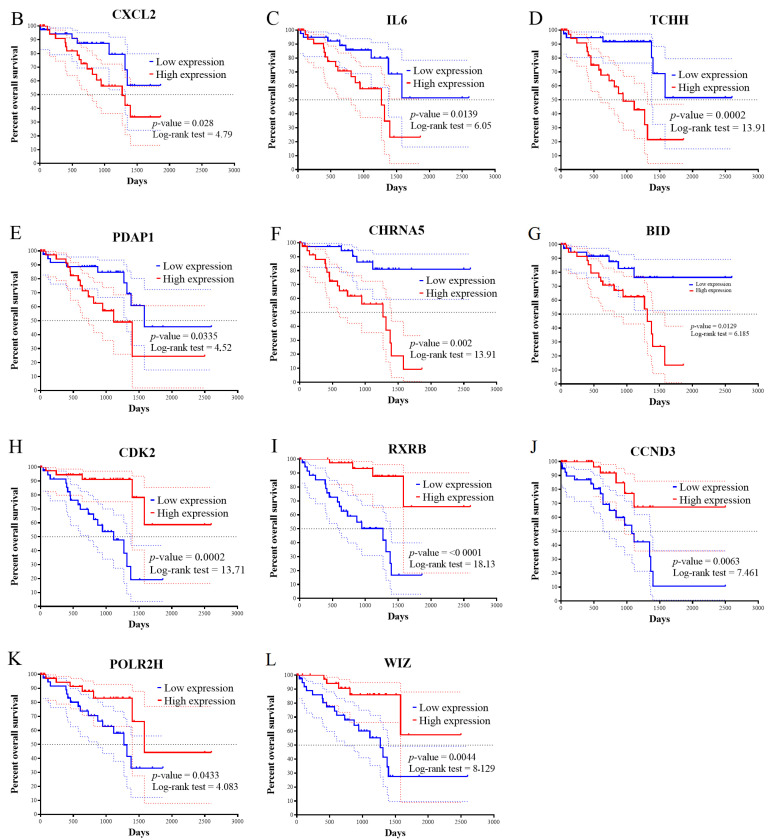
Survival analysis based on the prognostic model. (**A**). KM survival curve for the patients in the low- and the high-risk groups, the survival rate of patients in the high-risk group suffered a more drastic decrease. KM survival curves for the 11 genes of the prognostic model on the UVM TCGA cohort stratified by the gene median value of expression. Upregulation of CXCL2, IL6, TCHH, PDAP1, CHRNA5, BID (**B**–**G**) and downregulation of CDK2, RXRB, CCND3, POLR2H, and WIZ (**H**–**L**) were indicators of worse survival. The differences were statistically significant (*p* < 0.05).

Survival analysis was conducted to evaluate the prognostic model. The Kaplan–Meier (KM) survival curve for the low-risk and high-risk groups showed that patients in the high-risk group experienced a more significant decrease in survival rate (Figure 2A). Additionally, individual survival analyses were performed for each gene included in the prognostic model stratifying the UVM cohort by the median value of the gene expression (Figure 2B–L). The significant differences (*p* < 0.05) in survival outcomes observed upon upregulation of CXCL2, IL6, TCHH, PDAP1, and CHRNA5, and downregulation of CDK2, RXRB, CCND3, POLR2H, and WIZ in individual gene analyses suggest the potential of the gene expression profiles included in the model for personalized prognosis of UVM patients.

**Figure 3:**
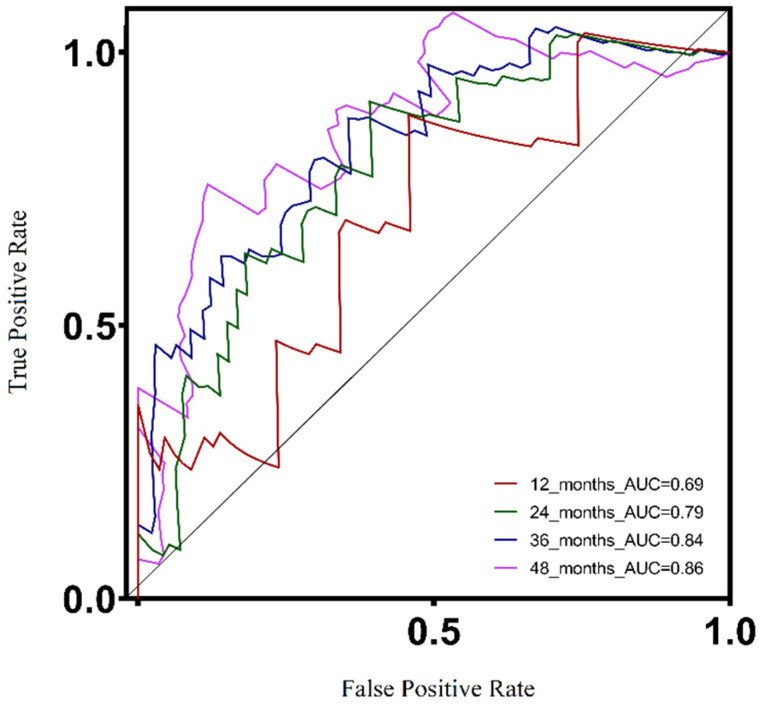
Accuracy of the prognostic model, ROC curve for overall patients’ survival of different years in the training cohort showed that the model had a potent predicting ability, with the 1-year, 2-year, 3-year, and 4-year AUC being 0.69, 0.79, 0.84, 0.86, respectively.

A ROC curve was used to demonstrate the prognostic ability of the UV response signature model in predicting patient survival based on gene expression data. The corresponding area under the curve (AUC) values for 1, 2, 3, and 4 years were 0.69, 0.79, 0.84, and 0.86, respectively (Figure 3). The ROC analysis suggested that the accuracy of the model increased progressively over time, as evidenced by the AUC values. These findings suggest that the UV response signature model could provide dependable long-term prognostic information for patients.

### 3.4. Functional Enrichment Analysis of UVM Patients by Their Risk Score

**Figure 4:**
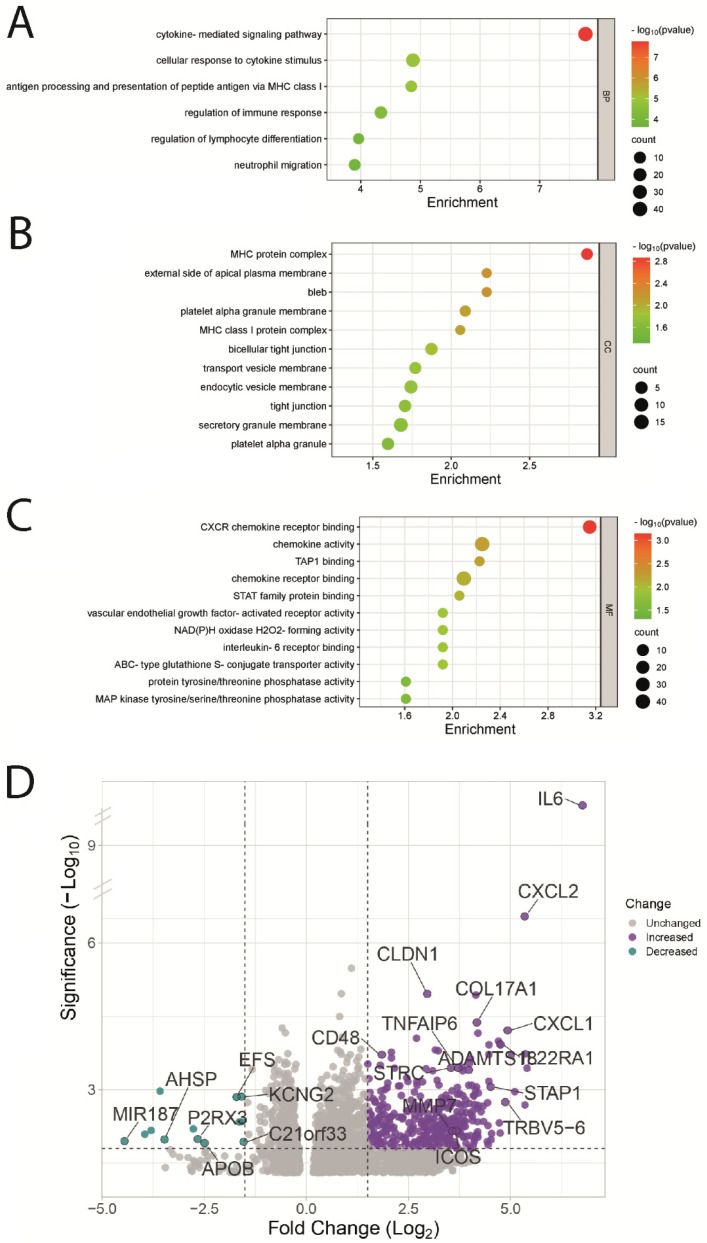
Functional enrichment and differential gene expression analysis. (**A–C**). GO Biological Process, GO Cellular Component, and GO Molecular Function Functional enrichment analysis for low-risk versus high-risk UVM patients. (**D**). Volcano plot showing selected differentially expressed genes between high and lowrisk UVM patients.3.5. Estimation of the Stromal and Immune Cells Infiltration Analysis.

GO enrichment analysis showed that several biological processes and molecular functions were enriched in differential genes between the high-risk and low-risk groups. These included cytokines signaling pathways, cellular response to cytokine stimulus, and regulation of immune response for biological processes, and chemokine receptor binding, chemokine activity, interleukin 6 receptor binding, and interleukin 1 receptor binding for molecular functions (Figure 4A–C). Moreover, the differential expression analysis revealed that immune-associated genes and extracellular matrix genes were upregulated in the high-risk group (Figure 4D). In our GSEA analysis, we conducted a principal component analysis (PCA) to assess the variance explained by different components in the defined high-risk and low-risk groups. The PCA figure revealed that PC1 accounted for 7.6% of the variance, PC2 accounted for 5.9% of the variance, and PC3 accounted for 3.3% of the variance (Supplementary Figure S1). In our GSEA analysis comparing uveal melanoma patients under high- and low-risk scores, we observed normalized enrichment scores (NES) for several biological processes. Specifically, we found notable enrichment in the inflammatory response, epithelial to mesenchymal transition, and interferon gamma response (Supplementary Figure S2).

### 3.5. Estimation of the Stromal and Immune Cells Infiltration Analysis

**Figure 5:**
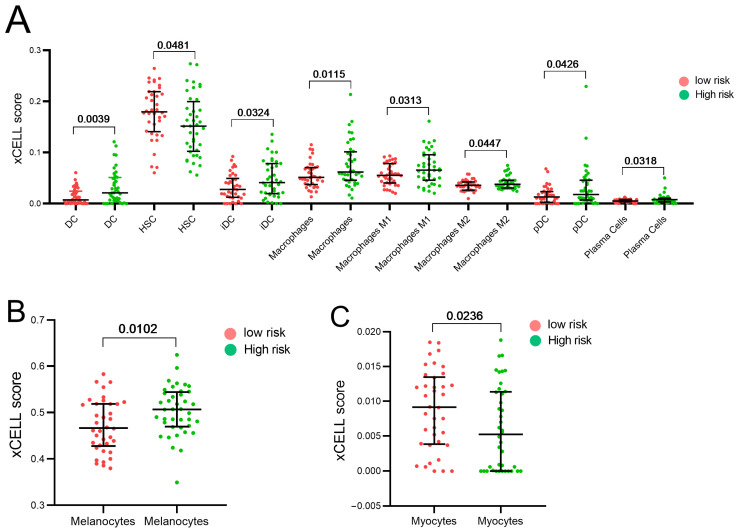
Estimation of immune and stromal cell infiltration in UVM patients. (**A**). Dispersion plots showed differential infiltration of immune cells between the low and high UVM risk groups, higher infiltration of dendritic cells (DC), immature dendritic cells (iDC), pan macrophages also M1 or M2 subtype, plasmacytoid dendritic cells (pDC), and plasma cells, were observed in the high-risk group (*p* < 0.05), whereas hematopoietic stem cells (HSC) were enriched in the low-risk group (*p* < 0.05). Differential stromal cell infiltration was observed between the low and high UVM risk groups. (**B**). Melanocytes were found to be overrepresented in the high-risk group. (**C**). Finally, myocytes were found to be enriched in the low-risk group compared to the high-risk group.

We used the “xCell” deconvolution algorithm to estimate the levels of infiltration of 64 immune and stromal cell lineages to investigate the relationship between tumor immune response and our risk model. Our results showed that the levels of immune infiltration were different between the two risk groups. Specifically, we found that the high-risk group had significantly higher levels of stromal cell infiltration, such as melanocytes and myocytes, as shown in Figure 5B,C. Furthermore, we observed that the high-risk group tended to have higher levels of immune cell lineages, including pan-macrophages and M2 polarized macrophages, dendritic cells (DC), immature dendritic cells (iDC), plasmacytoid dendritic cells (pDC), and plasma cells. In contrast, hematopoietic stem cells (HSC) were enriched in the low-risk group in the tumor microenvironment, as shown in Figure 5A.

### 3.6. Prognostic Model Genes Association with Clinicopathological Features of UVM Patients

**Figure 6:**
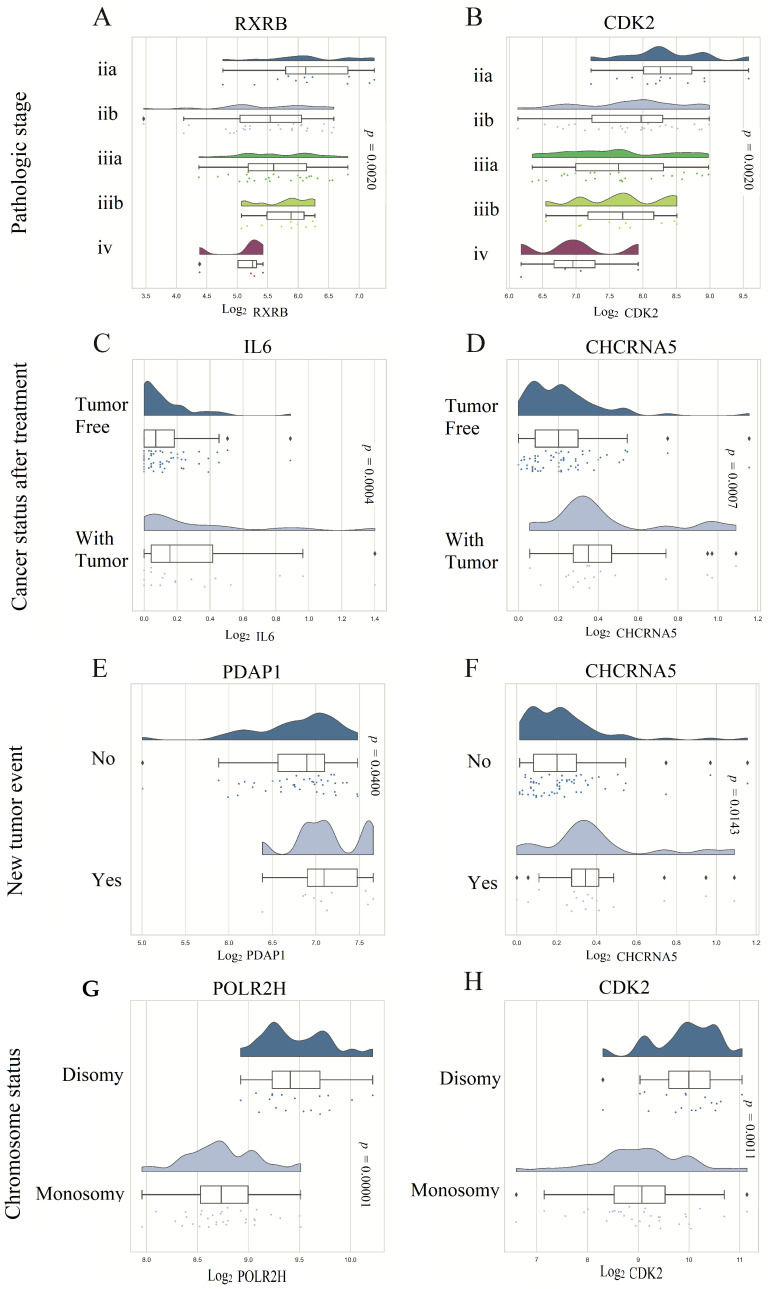
Association of 11 prognostic genes with clinicopathological features of UVM patients. Panels (**A**,**B**) show that RXRB and CDK2 expression levels were higher in lower pathological stages of the tumor. Panels (**C**,**D**) show that IL6 and CHRNA5 expression levels were lower in patients who were tumor-free after initial treatment. Panels (**E**,**F**) show that PDAP1 and CHRNA5 expression levels were higher in patients who presented a new tumor event after initial treatment. Finally, panels (**G**,**H**) show that POLR2H and CDK2 were upregulated in patients who had chromosomal disomies compared to those bearing chromosomal monosomies.

The analysis revealed that the expression levels of RXRB and CDK2 were higher in lower pathological stages of the tumor (Figure 6A,B). Conversely, the expression levels of IL6 and CHRNA5 were lower in tumor-free patients after initial treatment compared to those who still had remaining tumors (Figure 6C,D). Additionally, higher expression levels of PDAP1 and CHRNA5 were found in patients who experienced new tumor events after initial treatment (Figure 6D,E). Finally, POLR2H and CDK2 were found to be upregulated in patients with chromosomal disomies compared to those with chromosomal monosomies (Figure 6F,G).

### 3.7. Validation of the UV Response Gene Signature in the Context of a Metastatic UVM Cohort

**Figure 7:**
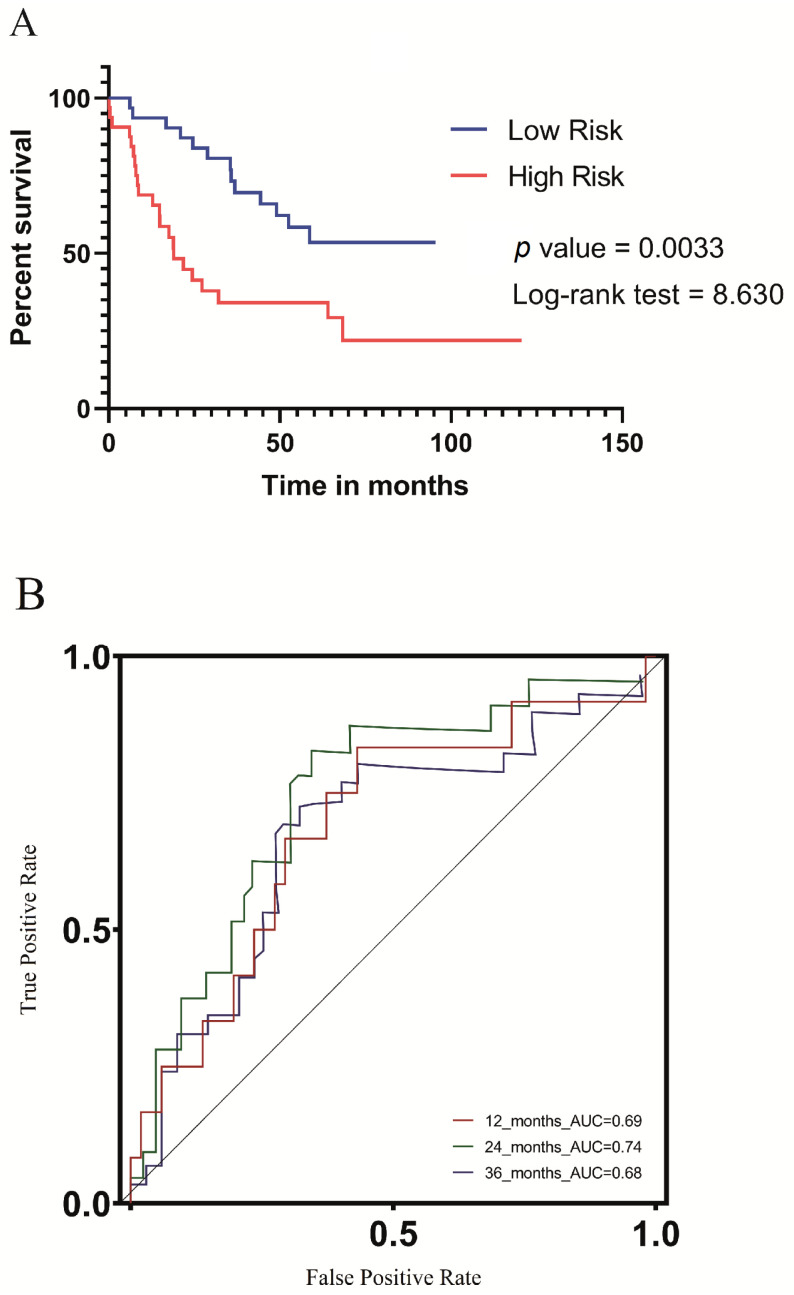
(**A**). Metastasis-free survival analysis based on the prognostic model in the GSE22138 validation cohort. A KM survival curve shows the patients in the low-risk (*n* = 31) and high-risk (*n* = 32) groups, indicating a more drastic decrease in survival rates for patients in the high-risk group. (**B**). The ROC curve of metastasis-free survival for different years in the validation cohort shows that the model had a potent predicting ability, with AUC values of 0.69, 0.74, and 0.68 for 1 year, 2 years, and 3 years, respectively.

During the validation phase, we assessed the prognostic performance of the UV light signature in the GSE22138 cohort [17]. We leveraged the ridge regression coefficients obtained from the TCGA training dataset, in which 11 genes were identified, and applied the formula outlined in Section 2.3 to compute a risk score for each patient in the validation cohort. We subsequently stratified the validation cohort into low-risk (*n* = 31) and high-risk (*n* = 32) groups based on the median risk score. Our analysis revealed that patients in the high-risk group had significantly worse metastasis-free survival (MFS), as illustrated in the KM survival curve (*p*-value = 0.0033) shown in Figure 7A. We validated the predictive performance of our UV response signature prognostic model for metastasis-free survival in an independent cohort. Our results indicate that the model exhibited a strong predictive ability, with AUC values of 0.69, 0.74, and 0.68 for 1, 2, and 3 years, respectively (Figure 7B). These findings suggest that our model can provide valuable prognostic information for patients with regard to their metastasis-free survival outcomes.

## 4. Discussion

The amount and quality of melanin in the eyes and skin provide varying levels of protection against ultraviolet radiation (UVR), which can cause physical or genomic damage to cells [18,19]. The eyes are at risk of oncogenic transformation into melanoma due to their exposure to UVR while producing visual input [10,20,21]. However, current classifications by the World Health Organization suggest that UVM is triggered by risk factors other than cumulative solar damage [9,22]. Nevertheless, evidence suggests that some UVMs possess molecular signatures reflective of UVR damage, even in those originating from the choroid, which is the most common UVM origin. This suggests that UVR in some cases, is involved in the etiology of UVM [18,23].

In this study, we developed a prognosis model based on the expression of genes associated with cellular UV response. The model allowed us to stratify patients into low- or high-risk score groups, our findings demonstrate a clear association between specific gene expression levels and adverse tumor outcomes. The forest plot in Figure 1B highlights that high expression levels of CXCL12, IL6, TCHH, PDAP1, CHRNA5, and BID are linked to an increased risk of poor outcomes, indicating a direct influence of these genes on tumor prognosis. Figure 2A further emphasizes the impact of gene expression levels on overall patient survival, reinforcing the potential of the genes in our model as prognostic markers for uveal melanoma. Our analysis of clinicopathological variables in Table 1 shows that the association between the altered genes and tumor outcomes is independent of traditional clinicopathological features. Although we observed one association with cytogenetic abnormality, no significant associations were found with other clinicopathological variables. This strengthens the notion that the influence of these genes on tumor outcomes is direct and not solely dependent on clinicopathological factors. Additionally, Figure 4 provides insights into the underlying biological processes associated with the altered genes in the high-risk group, aligning with known biological functions and pathways related to tumor progression. This concordance between gene expression and established biological processes further supports the causal relationship between altered genes and adverse tumor outcomes. Importantly, our model accurately predicted metastasis-free survival of UVM patients (Figure 7).

The identification of UV light response genes in UVM could be a valuable step toward understanding its pathogenesis. UVR has been identified as a potential risk factor for UVM development, and UV-induced DNA damage is considered an important event in UVM pathogenesis [8,24]. However, this is the first study to propose that a gene expression signature associated with UV light response could provide valuable insights into UVM prognosis. Additionally, understanding the mechanisms underlying the UV light response could lead to the development of novel therapies that target these genes and their pathways. Thus, the identification of UV light response genes holds the potential for improving the prognosis and treatment of UVM patients. However, it is important to acknowledge that the genes identified in our prognostic model can also be modulated by various environmental and cellular processes. For example, the gene CXCL2 can be influenced by other factors including viral infections [25]. Therefore, to accurately quantify the UV radiation exposure of UVM patients, it is crucial to conduct epidemiological studies that consider and account for UVR dosimetry and other potential modulating factors.

Our developed prognostic model demonstrates its capability to predict overall survival and metastasis-free survival in UVM patients, exhibiting a favorable performance with an area under the receiver operating characteristic curve (AUC) of 0.84 at 36 months (Figure 3). It is important to acknowledge the existence of alternative prognostic methods in the field. For instance, Cao et al. [26] proposed a pyroptosis gene signature-based prognostic model, achieving a ROC AUC of 0.88 for predicting five-year survival. Similarly, Cui et al. [27] developed a prognostic model utilizing autophagy signatures, which reached a ROC AUC of 0.91 for predicting one-year survival. In another study, Zhao et al. [28] applied a deep learning model focusing on the hypoxia phenotype, successfully predicting survival outcomes and tumor aggressiveness. By comparing our proposed model with these existing methods, we aim to underscore the potential of exploring gene expression data for predicting UVM patient survival. Moreover, we emphasize the significance of the cellular response to UV radiation as a potential mechanism in uveal melanoma.

In addition to the wide use of transcriptional signatures to predict the survival of patients with UVM, other layers of omics information have also been exploited. In one study, the authors conducted whole-genome sequencing of 103 UVM samples from different sites of the uveal tract was performed. Most UVM had a low tumor mutation burden (TMB), but two subsets with high TMB were identified: one driven by germline MBD4 mutation and another by ultraviolet radiation (UVR) exposure, which was restricted to iris UVM. All tumors had a known UVM driver gene mutation, and three other significantly mutated genes were identified (*TP53*, *RPL5*, and *CENPE*) [24]. The immune system has been extensively studied in the context of predicting the prognosis of UVM patients. Several studies have used gene expression profiles to identify immune cell subtypes and genetic markers associated with immunotherapy response. A six-immune cell signature was identified and used to determine three immune subtypes with significant differences in overall survival [29]. In addition, thirteen immune cells and one stromal cell were found to be significant in predicting poor overall survival rates in UVM patients, and a four-cell model was identified with the high-risk group being more sensitive to immunotherapy and chemotherapy [30]. Moreover, an immune and glycolysis six-gene signature was developed using LASSO and multivariate Cox regression analysis, which showed good predictive efficiency and was an independent risk factor for overall survival in UVM patients. Overall, these studies highlight the importance of the immune system and gene expression profiling in predicting the prognosis of UVM patients and identifying potential targets for therapy [31]. We contribute to the current knowledge by utilizing gene expression features of UV-responsive genes as a tool for prognosticating UVM patients (Figure 1B and Figure 2A).

There is little information regarding UV-responsive genes and their association with the progression of ocular tumors in general. One attempt was from the study of [32], the study aimed to investigate the role of UV radiation in the development of conjunctival malignant melanoma. The researchers analyzed six samples for mutations of the N-Ras gene, which is frequently found in cutaneous melanomas of sun-exposed areas. However, they could not detect any mutations, indicating that UV exposure may not be the cause of conjunctival melanoma development. In a seminal study, authors performed whole-genome sequencing on 103 UVMs from different sites in the eye and found that most have a low tumor mutation burden (TMB), but two subsets with high TMB were identified. One is driven by a germline MBD4 mutation, and the other is linked to UVR exposure, which is restricted to the iris. All but one tumor has a known UVM driver gene mutation, and three other significantly mutated genes (*TP53*, *RPL5*, and *CENPE*) were also identified. The limited therapeutic options for metastatic UVM have shown little impact, and these findings could provide insights into future treatment options, especially for high-risk subsets of UVM [24]. Despite not using genomic data from UVM patients in our study, we observed that the high-risk group showed an association with more cytogenetic abnormalities compared to the low-risk group. This suggests that the expression of UV-responsive genes could potentially be linked to the genomic instability of UVM tumors, as shown in Table 1.

The clinical challenges of UVM are complex and multifaceted. The tumor is often diagnosed at a late stage, and treatment options are limited. In addition, UVM is highly heterogeneous, and its molecular and genetic characteristics vary widely among patients [3]. One of the major clinical challenges in UVM treatment is the lack of effective systemic therapies. Although several treatments have been explored, such as chemotherapy, immunotherapy, and targeted therapy, most of them have shown no significant clinical benefit in randomized controlled trials [33,34]. With the exception of one study, which reported a median overall survival of 21.7 months for patients receiving tebentafusp, a bispecific antibody that activates T cells to target cancer cells, compared to 16 months for patients in the control group [35]. As such, there is still a significant unmet medical need for the development of innovative and effective therapies for UVM. In this sense, our results indicate that a higher infiltration of DC, iDC, pDC [36], and macrophages including M2 subtype [37], and plasma cells were observed in the high-risk group. Further research is warranted to understand the roles of these immune tumor microenvironment cells in UVM progression (Figure 5).

Despite the valuable insights gained from our study, it is important to acknowledge the limitations encountered. Firstly, the sample size of 80 UVM patients may be considered relatively small. However, uveal melanoma is a rare tumor with a low incidence in the general population, posing challenges in obtaining a large cohort. Nevertheless, the findings from our study contribute to the understanding of the molecular characteristics and prognostic factors of UVM. Another limitation pertains to the availability of complete clinicopathological information for all patients. In retrospective studies using existing datasets, obtaining comprehensive data for the entire cohort can be challenging. However, we made diligent efforts to maximize the available information and transparently reported any missing or unspecified data in our analysis. Furthermore, it is important to acknowledge inherent limitations in our study design our study focused solely on the analysis of gene expression and its association with prognosis, potentially excluding other important factors influencing tumor outcomes. Future studies should aim to address these limitations by incorporating larger patient cohorts, prospectively collecting comprehensive clinicopathological data, and considering additional factors that may impact tumor outcomes.

In summary, our study presents a perspective on the diagnosis, treatment, and prognosis of UVM, which holds potential for this type of tumor with a historically poor prognosis. The findings highlight the need for further investigation into the UVM tumor microenvironment, and preclinical research is necessary to expand the limited treatment options available to UVM patients.

## 5. Conclusions

The identification of genes responsive to UV light has implications for the prognosis of UVM. This knowledge could aid in developing personalized therapies that target the pathways of these genes. Further research in this area is warranted to fully comprehend the molecular mechanisms that underlie the response to UV light and its association with the pathogenesis of UVM.

## Figures and Tables

**Table 1:** Baseline clinicopathological data and correlations between risk score signatures.

Variable	All (*n* = 80)	Low Risk (*n* = 40)	High Risk (*n* = 40)	*p* Value
Age				
<60	36 (43.8%)	22	14	0.115 ^a^
≥60	44 (56.2%)	18	26	
TNM (Tumor/Node/ Metastasis)				
T2	4	4	0	0.068 ^b^
T3	36	15	21	
T4	38	21	17	
Missing data	2	0	2	
N0	76	39	37	0.304 ^b^
NX	4	1	3	
M0	73	38	35	0.482 ^b^
M1	3	1	2	
MX	4	1	3	
Pathologic stage				
IIA	4	3	1	0.376 ^b^
IIB	32	16	16	
IIIA	27	15	12	
IIIB	10	5	5	
IIIC	3	0	3	
IV	4	1	3	
Cytogenetic abnormality				
Chr 1 loss	3	2	1	0.042 ^b^
Chr 1 loss Chr 3 loss	2	0	2	
Chr 1 loss Chr 3 loss Chr 8q gain	4	0	4	
Chr 3 loss	2	1	1	
Chr 3 loss Chr 6p gain	1	1	0	
Chr 3 loss Chr 6p gain Chr 8q gain	5	3	2	
Chr 3 loss Chr 8q gain	18	8	10	
Chr 6p gain	11	9	2	
Chr 6p gain Chr 8q gain	5	5	0	
Chr 8q gain	1	1	0	
Missing data	28	10	18	
Gender				
Female	35	19	16	0.490 ^b^
Male	45	21	24	
Histological type				
Epithelioid Cell	13	5	8	0.322 ^b^
Epithelioid Cell Spindle Cell	21	9	12	
Spindle Cell	30	19	11	
Spindle Cell Epithelioid Cell	16	7	9	
Cancer status				
Tumor free	54	31	23	0.050 ^a^
With tumor	26	9	17	
Tumor basal diameter count				
From 7 to 16	27	10	17	0.174 ^a^
≥16	52	30	22	
Missing data	1	0	1	
Tumor tissue site				
Choroid	56	29	27	0.818 ^a^
Choroid Ciliary body	22	10	12	
Choroid Ciliary body Iris	2	1	1	

TNM tumor classification: T: primary tumor size and extent, higher numbers indicating increasing tumor size. The N: involvement of regional lymph nodes, higher numbers indicating increasing nodal involvement. M: metastasis, categorized as M0 (no distant metastasis), M1 (presence of distant metastasis), or MX (distant metastasis cannot be assessed). *p* values of the Fisher test (^a^), chi-square test (^b^).

## Data Availability

The article and Supplementary Material contain all the datasets produced for this study, and if there are any additional questions, they can be directed to the corresponding author.

## Supplementary Materials

The following supporting information can be downloaded at: https://www.mdpi.com/article/10.3390/biom13071148/s1, Supplementary Table S1: Univariate Cox regression of potentially prognostic UV response-related genes in UVM patients. Supplementary Figure S1: Principal component analysis (PCA) for uveal melanoma patients grouped as for the high- or low-risk group. Supplementary Figure S2: GSEA analysis comparing uveal melanoma patients under high- and low-risk scores.
