# Supplementary material for: Validation of an Ultraviolet Light Response Gene Signature for Predicting Prognosis in Patients with Uveal Melanoma
Source: Biomolecules. 2023 Jul 19;13(7):1148. doi: 10.3390/biom13071148 (PMC10377706; doi:10.3390/biom13071148)
Supplement: Supplementary file 1 [file biomolecules-13-01148-s001.zip › Supplementary Figure 1.pdf]

Supplementary Figure S1: Principal Component Analysis (PCA) for uveal melanoma patients grouped as for the high or the low risk group

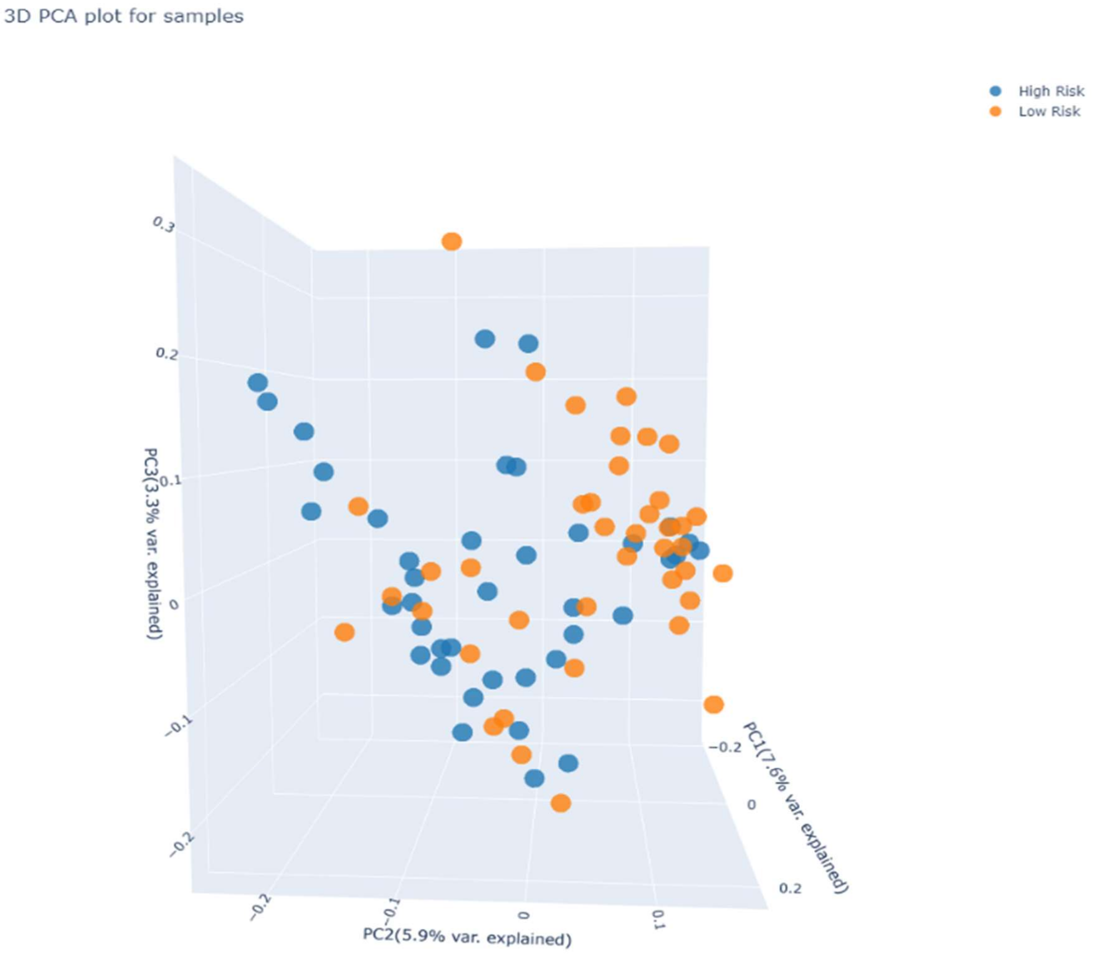

Supplementary Figure S1: We conducted a principal component analysis (PCA) to assess the variance explained by different components. The PCA figure revealed that PC1 accounted for 7.6% of the variance, PC2 accounted for 5.9% of the variance, and PC3 accounted for 3.3% of the variance. This indicates that these principal components capture a portion of the variability present in the dataset.
