# Supplementary material for: Validation of an Ultraviolet Light Response Gene Signature for Predicting Prognosis in Patients with Uveal Melanoma
Source: Biomolecules. 2023 Jul 19;13(7):1148. doi: 10.3390/biom13071148 (PMC10377706; doi:10.3390/biom13071148)
Supplement: Supplementary file 1 [file biomolecules-13-01148-s001.zip › Supplementary Figure 2.pdf]

Supplementary Figure S2: GSEA analysis comparing uveal melanoma patients under high and low risk scores

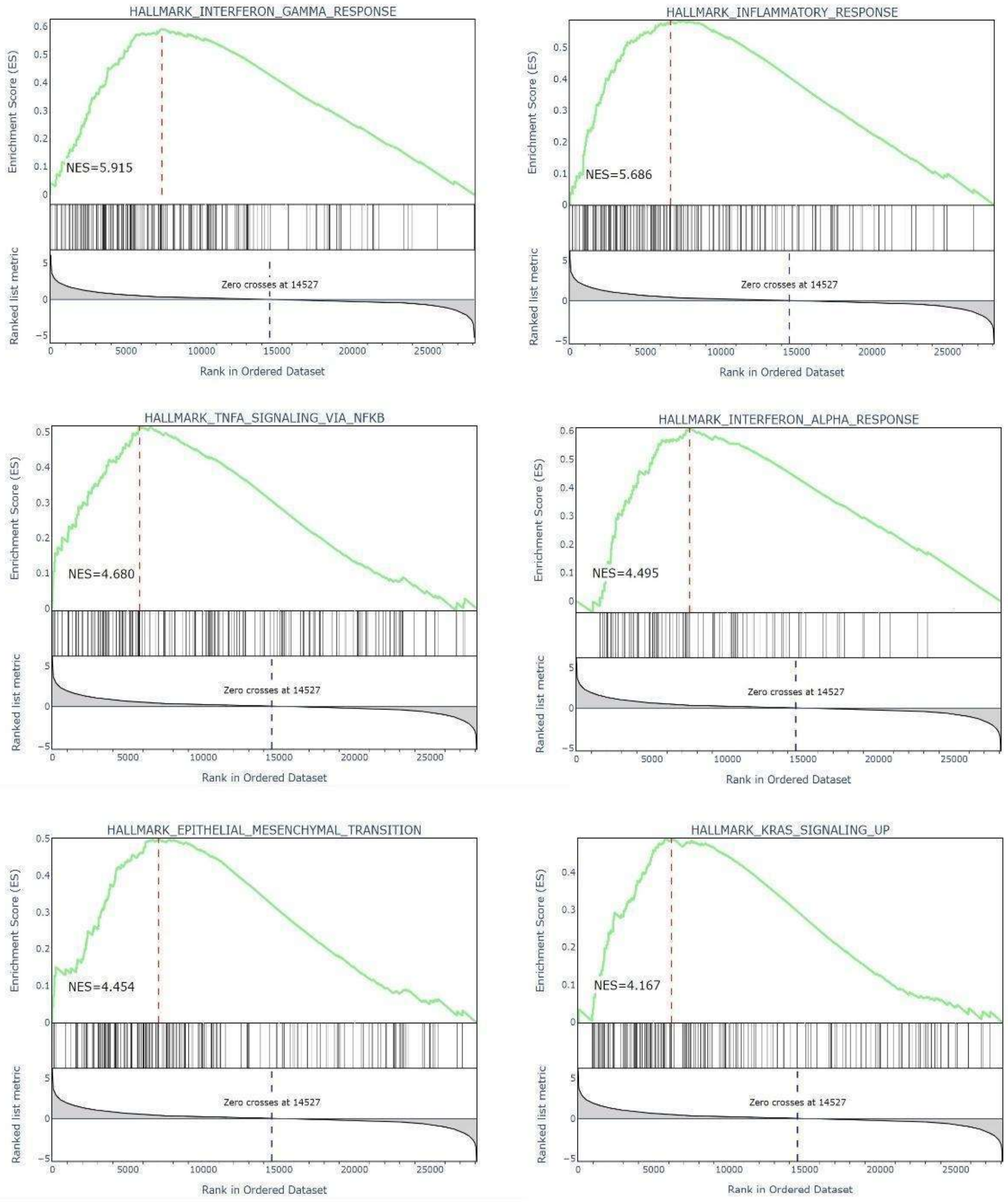

Supplementary Figure S2: GSEA analysis comparing uveal melanoma patients under high and low risk scores, we observed significant enrichment scores (NES) for several biological processes. Specifically, we found notable enrichment in the inflammatory response, epithelial to mesenchymal transition, and interferon gamma response. These findings suggest that there may be distinct molecular signatures associated with high and low-risk groups, potentially reflecting differences in immune activation, cellular plasticity, and response to interferon gamma signaling. These enriched biological processes shed light on the underlying mechanisms and pathways involved in uveal melanoma progression and may have implications for understanding disease heterogeneity and identifying potential therapeutic targets.
